# The Prevalence and Location of the Second Mesiobuccal Canals in Maxillary First and Second Molars Assessed by Cone-Beam Computed Tomography

**DOI:** 10.7759/cureus.24900

**Published:** 2022-05-11

**Authors:** Yousef Alnowailaty, Faisal Alghamdi

**Affiliations:** 1 Conservative Dentistry, King Abdulaziz University, Faculty of Dentistry, Jeddah, SAU; 2 Oral Biology, King Abdulaziz University, Faculty of Dentistry, Jeddah, SAU

**Keywords:** prevalence, saudi population, second mesiobuccal canal, maxillary molars, cone beam computed tomography

## Abstract

Objective

This retrospective study aimed to investigate the prevalence and location of the second mesiobuccal (MB2) canal in the mesiobuccal root of the maxillary first molar (MFM) and the maxillary second molar (MSM) through cone-beam computed tomography (CBCT) imaging.

Methods

One thousand two hundred CBCT images of the MFM and MSM were divided equally and analyzed. To standardize the methodology of MB2 detection, the observation and measurements were located at the pulpal floor by 1 mm apically. The distances between canals were calculated from the center point of the MB2 canal (PMB2) to the center point of both the first mesiobuccal canal (PMB1) and the palatal canal (PP). The data were provided in the form of frequencies and percentages. The chi-square test was used to analyze the differences among maxillary molars, while the significance level was set at 0.05.

Results

The prevalence of the MB2 canal in the MFM and MSM was 46.7% (p=0.020) and 17.7% (p=0.457), respectively. Additionally, the prevalence of the MB2 canal was more frequent in females. The average distance between PMB1 and PMB2 for MFM and MSM was 1.87±0.42 mm and 1.24±0.76 mm, respectively. Furthermore, the average distances of PMB2 and PP were 0.74±0.21 mm and 0.43±0.18 mm for MFM and MSM, respectively.

Conclusion

The MB2 canal was found in 386 (32.2%) of the maxillary molars. The Saudi people have a high possibility of discovering the MB2 canal. The CBCT is a useful and high-precision diagnostic tool not only for detecting but also for locating in vivo MB2 canal in the mesiobuccal root of maxillary molars.

## Introduction

The anatomy of the mesiobuccal (MB) root of maxillary molars has received the most attention in recent decades [1]. This root commonly has complex anatomy with two main root canals (called first mesiobuccal [MB1] and second mesiobuccal [MB2]) and loops, intercanal connections, auxiliary canals, and apical ramifications are all common anatomical features [2]. The orifice of the MB2 is often positioned within 3.5 mm palatally and 2 mm mesially of the MB1 and either mesial to or in the sub pulpal groove [3]. Moreover, It is frequently buried behind a dentine wall shelf or calcifications in a minor groove [4]. As a result, it is possible that it will be missed in normal clinical practice, especially if no magnification or special illumination equipment is used [5]. The challenging nature of locating this canal to effectively treat it has been identified as the primary cause of failure in maxillary molar root canal treatment [6-8]. Therefore, clinicians must be aware of MB2 prevalence and adopt procedural steps to locate and prepare it properly [3,5]. Several methodologies have been employed for determining the prevalence of the MB2 canal in maxillary molars, such as dye injection, sectioning, scanning electron microscopy, radiography, micro-CT or cone-beam computed tomography (CBCT) [9-12].

Intraoral radiographs remain the imaging modality of choice for pre-operative diagnosis, according to a joint position statement of the American Association of Endodontics and American Academy of Oral and Maxillofacial Radiology [13] and, more recently, an updated consensus of an expert committee convened by the European Society of Endodontology [14]. However, a small field of view (FOV) CBCT might be considered, for example, when complex anatomy is expected and for non-surgical retreatment of cases with possible missed canals. CBCT is indeed the gold-standard imaging tool for determining the existence of an MB2 canal in clinical preparation [11,15-17]. In observational studies, CBCT was already acknowledged as the most dependable device for application as it allows for consecutive evaluation of specific anatomical characteristics for all groups of teeth in greater populations [18].

Using CBCT, the average worldwide prevalence of MB2 was found to be 73.8%, with a range between 48% and 97.6% [16]. Studies reported the incidence of MB2 in the Saudi population to range between 23.3% and 86.8% in maxillary first molar (MFM) [19-24], while two studies found the incidence in maxillary second molar (MSM) between 19.8% and 80% [19,25]. The prevalence of MB2 in MFM and MSM in Riyadh, Saudi Arabia, was investigated in the first study by CBCT [19] and in the second study by micro-CT [25]. On the other hand, only one study investigated the prevalence of MB2 in MFM in the western Saudi Arabian subpopulation using CBCT [24]. Therefore, this study aims to determine MB2 incidence and location in MFM and MSM in a Saudi population considering gender and maxilla side (right/left side).

## Materials and methods

Sample selection

The study took place at the Oral and Maxillofacial Radiology Department of King Abdulaziz University (KAU) Dental Hospital (Jeddah, Saudi Arabia). Screening of 2,946 CBCT scans in a random fashion was performed. To rule out the impact of ethnic variations, Saudi citizens, who are residents of Jeddah city, were included in the sample. Furthermore, subjects had good quality CBCT scans and at least one MFM or MSM with a fully formed root. The excluded scans were either from non-Saudi citizens, low-quality CBCT images, the lack of at least one MFM/MSM or maxillary molars with developmental anomalies, root restoration, intracanal post, coronal restoration/ prosthetic crown, or open apex.

Only when indicated, CBCT was requested for treatment planning or diagnostic purposes for surgical, orthodontics, or endodontics cases. Demographic data such as gender, citizenship, and age were inquired with each scan. This investigation used a multi-stage stratified random sample with two database groups for CBCT images. After applying inclusion criteria, CBCT scans of 300 patients (mean±SD: 39.2±18.3) between the ages of 18 to 80 years, with equal numbers of both genders, collected between January 2013 and December 2021 were included from each database group in this study. The Research Ethics Committee at the Faculty of Dentistry of King Abdulaziz University (KAU) granted the ethical approval (approval no.: 354-12-21). The study was conducted in accordance with the Declaration of Helsinki. All participants signed an informed consent that their data will be anonymously used for research purposes according to the guidelines of the local ethics committee for this study.

Power analysis for sample size

An independent t-test was used in this investigation to calculate the power. For the provided values from the t-test with an alpha (α) level of 0.05 (5%), the power calculated was 0.86 with 1,000 subjects for the sample size for this study (500 patients per group for both genders). Power and Sample Size Calculation version 3.1.6 was used to estimate the sample size (PS software, Vanderbilt University, Nashville, USA).

Image evaluation

**Figure 1:**
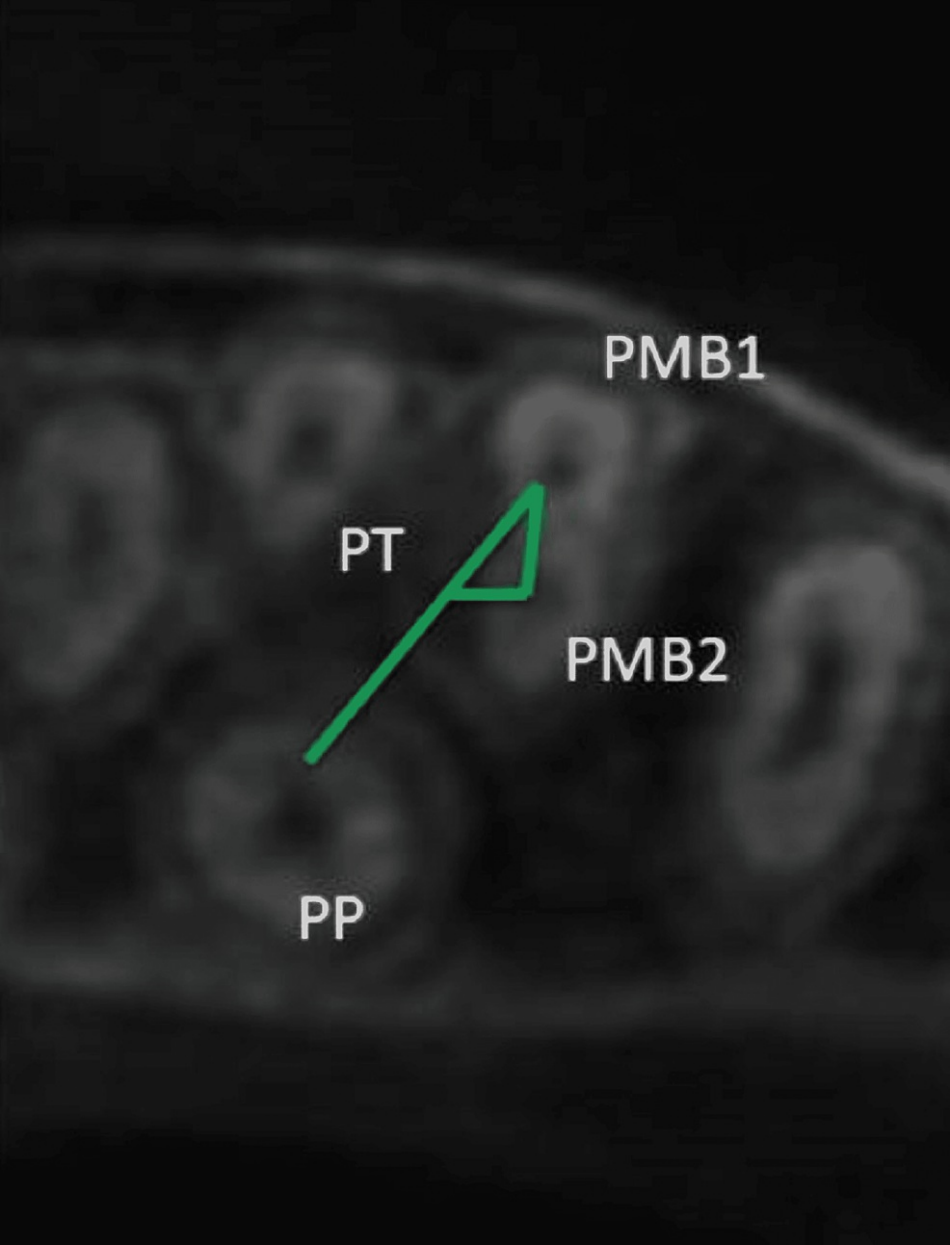
Axial view of the first molar on the right side of the maxilla Straight lines were constructed to connect the various points: the PMB1-PP line and the PMB1-PMB2 line. A third line, PMB2-PT, was drawn to represent a perpendicular line between PMB2 and the PMB1-PP line (PT point). The distance between the points was measured in millimeters using the lines drawn between them. PMB1 - first mesiobuccal canal center; PMB2 - MB2 canal center point; PP - center point of palatal canal; PT - perpendicular center point line between PMB2 and the PMB1-PP lines

Images were reconstructed and measured in the coronal, axial, and sagittal planes using OnDemand3D™ imaging software (Cybermed, Seoul, South Korea). Multiplanar reconstruction (MPR) was also employed for a detailed reflection of the root canal system. The MPR was taken in coronal-apical followed by apical-coronal directions. In the event of an unclear scan in either of the planes, the scanning was performed again, and the tooth was reexamined in a three-dimensional manner. The observation and measurements for standardizing the methodology of MB2 detection were located at the pulpal floor by 1 mm apically. The MB2 canal's geometric position in reference to the MB1 and the palatal canal (P) was identified. The central points of each canal (P) were located (PMB1, PMB2, and PP), and a straight line was projected between them (PMB1-PP and PMB1-PMB2). A third line was drawn (PMB2-PT) perpendicular to the PMB1-PP line (PT point) based on a protocol outlined in three previous studies [26-28]. The distances of the lines drawn between the points were measured in millimeters (see Figure 1). The outcome variables observed were the number of MFM and MSM and the prevalence of the MB2 canal in the MB roots projected into three planes (axial, sagittal, coronal) while taking into account the right or left side in the same patient. Two experienced endodontists (authors of the article) have CBCT image scanning and evaluation experience between five and seven years. Both authors inspected the scans and determined the number of MFM and MSM along with the prevalence of MB2 canal in MB root of those molars considering the maxilla side (right/left side) in the same patient. The location of MB2 canals and their distances in MFM and MSM was also calculated. A hundred random CBCT scans that were not included in this study were assessed by both authors and one consultant radiologist for standardization purposes. The calibration process was performed at two different points in the study with an interval of two weeks. The presence of a consultant radiologist was required for the standardization of CBCT scan assessment.

Statistical analysis

The gathered data was analyzed using SPSS Version 20.0 for Windows (IBM Corp., Armonk, USA). Inter- and intra-examiner reliability for CBCT scan interpretation was assessed using Cohen's kappa test. The data was given as frequencies and percentages. The relevancy between gender and maxilla side in relation to the presence/absence of MB2 in MFM and MSM was determined by using a chi-squared test. The average distances between the points PMB1-PMB2, PMB1-PP, and PMB2-PT, were calculated using 95% confidence intervals (CI). The statistical significance level was determined at 0.05.

## Results

Inter- and intra-examiner reliability

There was an almost inter-examiner agreement regarding the presence/absence of MB2 in MFM (kappa≥0.93), MB2 in MSM (kappa≥0.98), and location of MB2 detection (kappa≥0.96). For intra-examiner reliability, the agreement of the two examiners with regards to the presence/absence of MB2 in MFM was kappa≥0.97 and kappa≥0.93, for MB2 in MSM was kappa≥0.94 and kappa≥0.94, and for the location of MB2 detection was kappa≥0.98 and kappa≥0.98), respectively.

Prevalence of MB2 canal in maxillary first and second molars

The overall number of screened MFM and MSM of 300 CBCT images was 1,200 teeth (MFMs=600, MSMs=600). The overall prevalence of MB2 canals among MFM and MFM of 300 CBCT images was 32.2% (n=386 out of 1,200) (MFMs=280, MSMs=106; see Table 1). Although there was a statistically significant association between total MB2 canals detected and gender (p=0.048), with a higher prevalence of MB2 canals in females (34.5%) when compared to the male (29.8%, p=0.048). Overall, the highest prevalence of the MB2 canal was on the left side (33.7%) when compared to the right side of the maxilla (30.7%, p=0.147; see Table 1).

**Table 1:** The general distribution of the number of second mesiobuccal canals in all the maxillary first and second molars by gender and side MB2 - second mesiobuccal canals

Number of MB2 canal in all maxillary 1^st^ and 2^nd^ molars		MB2 in all maxillary 1^st^ and 2^nd^ molars		Total (100%)	Chi-square	p-value
		Present (%)	Absent (%)			
Gender	Male	179 (29.8%)	421 (70.2%)	600	2.994	0.048
	Female	207 (34.5%)	393 (65.5%)	600		
	Total	386 (32.2%)	814 (67.8%)	1200		
Side	Right side	184 (30.7%)	416 (69.3%)	600	1.237	0.147
	Left side	202 (33.7%)	398 (66.3%)	600		
	Total	386 (32.2%)	814 (67.8%)	1200		

Prevalence of MB2 canal in maxillary first molars

The MB2 canal was detected in 46.7% of the analyzed cases (280/600; Table 2). Regarding the percentage distribution of the MB2 canal based on the side, a statistically significant difference was observed (p=0.020): 45.3% on the left and 48.0% on the right side (Table 2). Moreover, when the prevalence of the MB2 canal in relation to gender was evaluated, a statistically significant difference was noted (p=0.020), with 51.0% in females and 42.3% in males (Table 2). The distances between the points were calculated with a 95% confidence interval. The distance between PMB1-PP was 5.04±1.17 mm. For PMB1-PMB2, the average distance was 1.87±0.42 mm, and it was 0.74±0.21 mm for PMB2-PT.

**Table 2:** The distribution of the number of MB2 in maxillary first molars by gender and side MB2 - second mesiobuccal canals

Number of MB2 canal in maxillary 1^st^ molars		MB2 in 1^st^ molars		Total (100%)	Chi-square	p-value
		Present (%)	Absent (%)			
Gender	Male	127 (42.3%)	173 (57.7%)	300	4.527	0.020
	Female	153 (51.0%)	147 (49.0%)	300		
	Total	280 (46.7%)	320 (53.3%)	600		
Side	Right side	144 (48.0%)	156 (52.0%)	300	4.527	0.020
	Left side	136 (45.3%)	164 (54.7%)	300		
	Total	280 (46.7%)	320 (53.3%)	600		

Prevalence of MB2 canal in maxillary second molars

The MB2 canal was detected in 17.7% (106/600) of the cases (Table 3). When a comparison between the number of the MB2 canals on the left side (22.0%) versus the right side (13.3%) was made, there was no statistically significant difference (p=0.457; Table 3). There was no statistical significance (P= 0.457) according to gender, but the representation of the MB2 canals was slightly more frequent in females (18.0%) than in males (17.3%; Table 3). The distances between the points were calculated with 95% confidence intervals. The distance between PMB1-PP was 4.92±1.34. For PMB1-PMB2, the average distance was 1.24±0.76 mm, and it was 0.43±0.18 mm for PMB2-PT.

**Table 3:** The distribution of the number of second mesiobuccal canals in maxillary second molars by gender and side MB2 - second mesiobuccal canals

Number of MB2 canal in maxillary 2^nd^ molars		MB2 in 2^nd^ Molars		Total (100%)	Chi-square	p-value
		Present (%)	Absent (%)			
Gender	Male	52 (17.3%)	248 (82.7%)	300	0.046	0.457
	Female	54 (18.0%)	246 (82.0%)	300		
	Total	106 (17.7%)	494 (82.3%)	600		
Side	Right side	40 (13.3%)	260 (86.7%)	300	0.046	0.457
	Left side	66 (22.0%)	234 (78.0%)	300		
	Total	106 (17.7%)	494 (82.3%)	600		

## Discussion

The MB2 canal prevalence was assessed in MFM and MSM using CBCT scans in one hospital in Jeddah city, Saudi Arabia, for this retrospective study with a cross-sectional design. This type of work presents the data on MB2 canal prevalence at a specific period in time.

Since its recent introduction in dentistry, CBCT has been commonly used for a variety of reasons, including endodontic diagnosis. Although CBCT might be beneficial in endodontic diagnosis, there is little information on the influence of obturation material, voxel resolution, and professional experience on the agreement for MB2 canal observation [29,30]. A study carried out in 2015 concluded that agreement during MB2 inspection is more influenced by the root canal filling and examiner experience than voxel size [31]. Moreover, another study described a comparable accuracy of MB2 canals detection between CBCT scans and clinical sectioning [32]. Despite the fact that CBCT scans do not offer 100% precision, they are an additive to the endodontic analytical tools, particularly in nonsurgical retreatment where the MB2 canal was initially missed [33].

A recent systematic review and meta-analysis illustrated that the prevalence of the MB2 canal in MFM was looked at in 22 investigations (41 populations) with a pooled incidence of 69.6% (64.5%-74.8%). MB2 in MSM was identified in 16 investigations (17 populations) with a pooled incidence of 39.0% (31.1%-46.9%). The prevalence of MB2 in MFM was significantly higher compared to the MSM (p=0.000) [18]. These findings agree with our study that showed the prevalence of the MB2 canal was higher in MFM (46.7%) than MSM (17.7%).

Several studies found that the prevalence of the MB2 canal in MFM using CBCT ranges between 19.65 and 89.5%, depending on the ethnic groups. East Asian regions have been associated with the highest occurrence of MB2 canal - in Korea (63.6% - 71.8%) [15,33], China (30.9% - 69.4%) [34-36], and Taiwan (45.9%) [37]. In Southern Asia, the highest incidence was found in the Thai population (63.6%) [38], followed by India (49%) [17]. In Africa, the frequency of MB2 was 89.5% among South African populations [39]. Previous investigations in Europe have found MB2 prevalence of 86.2%, 71.3% and 59.5% in Spanish [40], Portuguese [41], and Polish populations [42], respectively. In an American study, the prevalence of MB2 in initial treatment was 61.9% [43]. Furthermore, the prevalence of MB2 ranged between 44.0 and 88.5% in the Brazilian population [11,44,45] compared to a range between 69.8% and 73.4% in the Chilean population [28,46]. Studies from different Middle Eastern countries have found percentages ranging between 46% and 70.2% in Iranian populations [47-49], 19.65% in Turkish [50], and 74.5% in Egyptian [51] populations.

The prevalence of MB2 canal in maxillary second molars was 17.7% in the current study, which is deemed low, and contradicts with the outcomes reported for Korean populations (34.4% -42.2%) [15, 33], Taiwanese population (32.3%) [37] and Chinese population (between 13.45 - 28.0%) [34-36]. In Southern Asia, the highest incidence was found in the Indian population (38%) [17], followed by the Thai population (29.4%) [38]. In Africa, the frequency of MB2 was 67.0% among South African populations [39]. Previous investigations in Europe have found a frequency of 47.3%, 43.8% and 23.2% in Spanish [40], Portuguese [41], and Polish populations [42], respectively. In an American study, the prevalence found in initial treatment was 37.7% [43], whereas, in the Brazilian population, the prevalence ranged between 21.9 and 83.4% [11,44,45]. In the Chilean population, the prevalence ranged between 42.5 and 46.9% [28,46]. Studies from different Middle Eastern countries have found percentages ranging between 14 and 43.4% in Iranian populations [47-49], while in Turkish [50] and Egyptian [51] populations were 17.7% and 57.9%, respectively.

Only one investigation had previously looked into MB2 using CBCT among maxillary first and second molars in a Saudi population [19]. This study concluded the prevalence of MB2 in MFM and MSM by 51.3% and 19.8%, respectively [19]. These percentages were higher than the present study findings in MFM and MSM by 46.7% and 17.7%, respectively.

A recent meta-analysis included 38 studies (58 population groups) that analyzed the association between gender and the occurrence of the MB2 canal in maxillary molars. This meta-analysis showed considerably greater odds of having the MB2 canal in males compared to females (p<0.05) [18]. This contrasts with our findings, which showed that the MB2 canal in maxillary molars occurred more commonly in females than males (p=0.048). Contradictory to what was reported by Betancourt et al. [52] and Zheng et al. [12], our findings suggested a statistically significant difference regarding the gender correlation with the maxillary molars.

In our study, the MB2 canal showed a high tendency to appear bilaterally (97.14%, n=272 out of 280) in MFM and (75.47%, n=80 out of 106) in MSM. The overall prevalence of the bilateral MB2 canal was 91.2% (352/386). Different studies reported similar findings regarding the bilateral MB2 canals in maxillary molars [15,28,37,38,47,50]. This indicates that if there is an MB2 canal on one side, there is a high chance that it exists in the contralateral mesiobuccal root.

Only in vitro studies have been used to determine the geometrical position of the MB2 canal [3,26,53,54]. However, a previous study by Betancourt et al. [28] demonstrated the efficiency of CBCT in locating the MB2 canal in vivo. In the MFM, the MB2 canal was located 2.68±0.49 mm palatally and 1.25±0.34 mesially to the MB1 canal. In the MSM, it was positioned at 2.41±0.64 mm palatally and 0.98±0.33 mm mesially. In another investigation by Betancourt et al. [27] applying the same method, it was found to be 2.2±0.54 mm palatally and 0.98±0.32 mesially to the MB1 canal in the MSM. In addition, Magat et al. [50] found the average distance between PMB1-PMB2 was 2.95±0.58 mm, 3.08±0.67 mm for MFM and MSM, respectively. For MFM and MSM, the average PMB2-PP distance was 5.81±1.09 mm and 5.55±1.09 mm, respectively. Görduysus et al. [3] reported that the MB2 can be found at 1.65±0.72 mm palatally and at 0.69±0.42 mesially to the MB1 canal regarding the MFM with MSM together.

Our findings for the location of the MB2 canal are different than excepted findings of previous studies using different techniques such as CBCT [28,50], stereomicroscope [54], and scanning electronic microscopy [55]. This might be attributed to the great sensitivity of in vitro studies contrarily to the use of microscopes at different magnifications that distort images. The resolution of the generated picture from CBCT is isotropic, i.e., the smallest unit of data, voxels. It is equivalent to measurements along three spatial axes, allowing images to be obtained without magnification and distortion (1:1).

Changes in the geometrical position of the MB2 canal towards the mesial or palatal side with respect to the MB1 canal may depend on the type of investigation. Arch anatomical relationships and proportions are lost in in vitro studies contrarily to CBCT, where all axes and planes can be observed [28]. These findings suggest that CBCT is a useful, high-precision diagnostic method for identifying and locating the MB2 canal in the mesiobuccal root of maxillary molars in vivo, enhancing the chances of successful endodontic treatment. The method applied in this work reveals that the geometric position of the MB2 canal in vivo is attainable. However, CBCT can identify and map the mesiobuccal root canal system, potentially improving the quality of endodontic therapy [24].

Study strengths and limitations

This study evaluated all of the obtainable CBCT scans of MFM and MSM that meet the inclusion criteria. To our knowledge, this is the first retrospective study with a cross-section design that has addressed the topic of MB2 prevalence among Saudi people in Jeddah city in-depth. The study included 300 CBCT images taken between 2013 and 2021 for variable medical/dental reasons. The CBCT scans were obtained from one hospital. Therefore, possible biases such as variances in exposure duration and setting were avoided. Regarding the limitations of the study, it attempted to analyze the MB2 location and prevalence in a small number of cities around educational dental hospitals. The individuals come from various locations, but they don't reflect the entire Saudi population. The information on the location of MB2 regarding the suitable technique to calculate is different from one study to another [28,56].

## Conclusions

According to this retrospective study, the Saudi people have a higher possibility of discovering the MB2 canal in MFM than MSM. The overall prevalence of the bilateral MB2 canal was 91.2%. To locate the geometric position of the MB2 canal at the mesiobuccal root, we propose to use the center of the main mesiobuccal canal as a reference parameter. Knowing about this anatomic variance beforehand aids a clinician in locating and treating all canals. Based on the findings, it is critical to investigate the presence of the MB2 canal for the best outcomes in the treatment of maxillary first and second molars. In order to locate the MB2 canal, the design of the access cavity must be changed from triangular to rhomboidal, use of ultrasonic, magnification, and, if required toughening of the pulpal floor to a depth of around 2 mm. Based on the results of this study, it's recommended to consider CBCT as an additional diagnostic method before starting root canal treatment of maxillary molars to obtain optimal results.
